# Polyclonal Hyperviscosity Crisis and Severe Depletion Coagulopathy Induced by Therapeutic Plasma Exchange in Sjögren’s Syndrome: A Case Report and Therapeutic Dilemma

**DOI:** 10.3390/reports9030207

**Published:** 2026-07-01

**Authors:** Gabriela Rybka, Andrzej Boryczko, Radosław Dziedzic, Łukasz Chmura, Joanna Kosałka-Węgiel

**Affiliations:** 1 Clinical Department of Rheumatology, Immunology and Internal Medicine, University Hospital in Kraków, Jakubowskiego 2, 30-688 Kraków, Poland; rybka.gabriela@gmail.com (G.R.); andrzej.boryczko@uj.edu.pl (A.B.); radoslaw.dziedzic@doctoral.uj.edu.pl (R.D.); 2 Doctoral School of Medical and Health Sciences, Jagiellonian University Medical College, Św. Łazarza 16, 31-530 Kraków, Poland; 3 Department of Pathomorphology, Faculty of Medicine, Jagiellonian University Medical College, Grzegórzecka 16, 31-531 Kraków, Poland; lchmura@su.krakow.pl; 4 Department of Rheumatology and Immunology, Jagiellonian University Medical College, Jakubowskiego 2, 30-688 Kraków, Poland

**Keywords:** cryoglobulinemia, hyperviscosity syndrome, plasmapheresis, Sjögren’s syndrome, rituximab

## Abstract

**Background and Clinical Significance:** Hyperviscosity syndrome (HVS) is a rare complication of primary Sjögren’s syndrome (pSS). While therapeutic plasma exchange (TPE) is the standard treatment to clear pathogenic immunoglobulins, its execution can trigger severe, atypical systemic risks. **Case Presentation:** A 60-year-old woman with pSS and extreme polyclonal hypergammaglobulinemia (total protein 100 g/L, IgM 41 g/L) presented with an acute hyperviscosity crisis, causing retinopathy, neurological deficits, and skin ischemia. Emergency TPE with 5% albumin replacement successfully reduced IgM by ~90% (to 6.39 g/L), resolving HVS symptoms. However, 20 min post-procedure, the patient suffered sudden hemodynamic collapse (BP 50/30 mmHg) and developed multiple massive, expanding soft-tissue hematomas. Laboratory tests revealed a coagulopathy consistent with plasma protein depletion following therapeutic plasma exchange, characterized by severe hypofibrinogenemia (1.35 g/L) and a 50% reduction in total serum protein. TPE was permanently discontinued. The patient was successfully stabilized using aggressive fluid resuscitation, vasopressors, and fresh frozen plasma (FFP) transfusions, followed by maintenance therapy with rituximab. **Conclusions**: In conclusion, clinicians should remain vigilant that severe hyperviscosity syndrome can be driven by a polyclonal increase in immunoglobulins rather than just monoclonal entities; furthermore, managing this condition requires careful balancing of TPE efficacy against its potential to trigger profound depletion coagulopathy.

## 1. Introduction and Clinical Significance

Cryoglobulinemia is characterized by the presence of immunoglobulins that precipitate at temperatures below 37 °C, leading to a spectrum of clinical manifestations ranging from mild purpura to life-threatening organ involvement [1,2]. Hyperviscosity syndrome (HVS), most frequently observed in monoclonal (type I) cryoglobulinemia but also possible in mixed forms, results from markedly increased concentrations of circulating immunoglobulins and/or cryoglobulin-containing immune complexes and can present with neurologic deficits, renal impairment, and cutaneous ischemia [1,2]. The pathogenesis involves both quantitative and qualitative alterations in circulating cryoglobulins, with hemodynamic consequences that may rapidly progress to end-organ damage [3,4,5].

The aim of this report is to present a case of successful plasmapheresis use in the treatment of cryoglobulinemia-associated hyperviscosity syndrome and to emphasize its role in the current therapeutic algorithm.

## 2. Case Presentation

**Figure 1:**
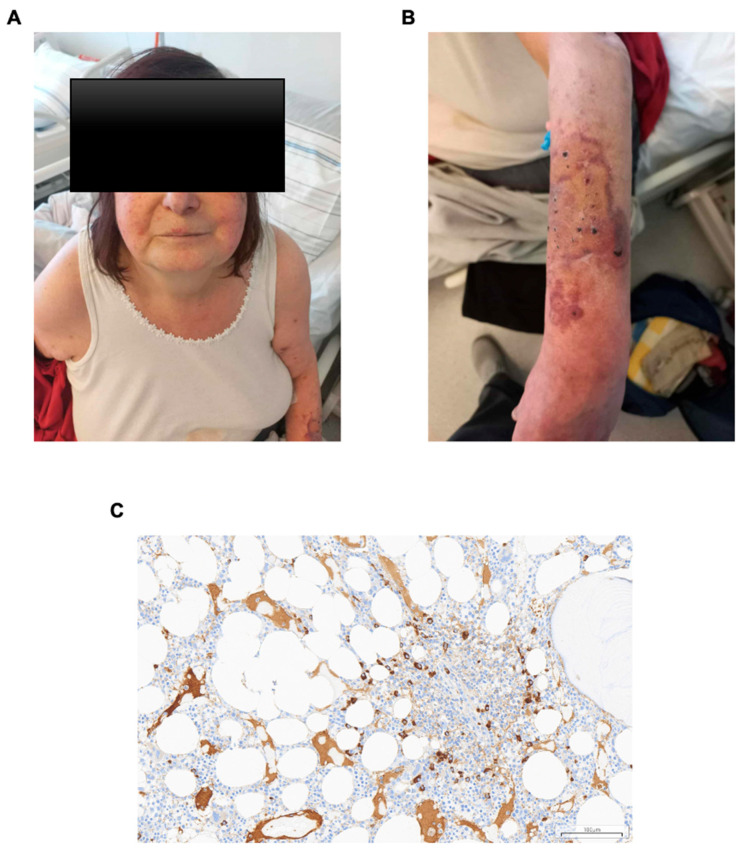
(**A**) Facial changes (petechial purpura); (**B**) changes on the upper extremities (distal extremity cooling and cyanosis, along with livedo reticularis); (**C**) trephine biopsy from the iliac bone. The biopsy shows a population of monotypic plasma cells (kappa+, IgM+). Whole-slide digital image; approximate equivalent of a ×20 objective (×200 conventional total magnification); scale bar = 100 µm.

A 60-year-old woman with a 12-year history of primary Sjögren’s syndrome (pSS) was treated with systemic glucocorticosteroids (sGCSs) and chloroquine. The diagnosis of pSS was established in 2012 based on strongly positive antinuclear antibodies (ANA > 1:10,240) and elevated rheumatoid factor (RF). In 2016, the patient developed interstitial nephritis accompanied by marked polyclonal hypergammaglobulinemia, which responded well to treatment with sGCSs and chloroquine. However, chloroquine therapy was later discontinued because of drug-induced maculopathy, and the patient remained on maintenance methylprednisolone therapy at a dose of 4 mg/day. Although polyclonal hypergammaglobulinemia was well-documented, routine screening for circulating cryoglobulins or serum viscosity was omitted during follow-up due to the complete absence of classic small-vessel vasculitis features (e.g., palpable purpura, digital ulcers). Other comorbidities were unremarkable. She was urgently referred to the Department of Rheumatology and Immunology due to new-onset headache, lower extremity pain, Raynaud’s phenomenon, reduced exercise tolerance, recurrent epistaxis, and facial skin changes (Figure 1A). On admission, physical examination revealed a petechial rash on the upper extremities, more pronounced on the left side, accompanied by distal extremity cooling and cyanosis, along with livedo reticularis (Figure 1B). Initial laboratory tests were complicated by rapid blood clotting. Results showed anemia (Hb 10.1 g/dL; RR: 12.0–16.0) without hemolysis (normal LDH and bilirubin), and normal platelet count. Coagulation parameters including activated partial thromboplastin time (aPTT) and international normalized ratio (INR) were within normal limits. Creatinine level was elevated at 131.8 µmol/L (RR: 44.0–80.0) with reduced eGFR (38 mL/min/1.73 m^2^). Urine sediment was unremarkable. Further tests revealed marked hypergammaglobulinemia (60.40 g/L; RR: 6.40–16.20), positive cryoglobulins, and significantly elevated immunoglobulins (IgG 40 g/L, IgM 41 g/L, IgA 23 g/L) along with raised serum free kappa light chains (222 mg/L; RR: 3.30–19.4). Lambda light chains were normal. The very high total protein prevented exclusion of a monoclonal component by immunofixation electrophoresis. A qualitative assessment performed on 8 April 2024 formally confirmed the presence of circulating cryoglobulins. Quantitative measurements of serum viscosity and cryocrit percentage were not performed, as these specific laboratory methodologies were unavailable at our center at the time of the patient’s acute hospitalization. Ophthalmologic exam revealed retinopathy consistent with hyperviscosity syndrome. To systematically evaluate the hematological baseline, a structured differential diagnostic process was executed to distinguish secondary autoimmune cryoglobulinemia from an underlying lymphoproliferative malignancy. Whole-body computed tomography (CT) ruled out overt lymphoma, demonstrating no lymphadenopathy or hepatosplenomegaly. The bone marrow trephine biopsy revealed a sparse population of monotypic (kappa+, IgM+) plasma cells but lacked the hypercellularity or architectural distortion pathognomonic for Waldenström macroglobulinemia. Concurrently, a rigorous infectious disease workup was performed to screen for secondary triggers of mixed cryoglobulinemia. Serological and molecular assays for Hepatitis B Virus (HBV), Hepatitis C Virus (HCV), Human Immunodeficiency Virus (HIV), and Epstein–Barr Virus (EBV) were uniformly negative, effectively ruling out chronic viral infections as drivers of the immune-complex formation. Histology was consistent with either IgM MGUS or cold agglutinin disease. Based on Ferri’s criteria and clinical signs, a diagnosis of cryoglobulinemia with hyperviscosity syndrome was established [1,2,3,4,5]. A total of three therapeutic plasma exchange (TPE) sessions were performed via a temporary dialysis catheter. The first session involved a 2000 mL plasma volume exchange, which was increased to 2500 mL for the two subsequent sessions. Volume replacement was achieved using a 5% human albumin solution, avoiding fresh frozen plasma and 20% albumin. Anticoagulation was maintained with unfractionated heparin (4000 IU bolus, followed by 3000 IU/hour). Intra-procedural management included vital signs monitoring and the administration of 40 mg methylprednisolone, calcium chloride, magnesium sulfate, and potassium chloride to prevent electrolyte depletion. Following the third TPE session, the patient experienced a sudden hemodynamic collapse approximately 20 min after returning to the ward, without loss of consciousness or head injury. Physical examination revealed critical hypotension, with blood pressure (BP) dropping to 50/30 mmHg (heart rate 90 bpm, SaO_2_ 92%). Concurrently, bleeding complications emerged, manifesting as a localized left trochanteric hematoma later that evening and progressing to multiple massive soft-tissue hematomas on both arms and the left hip by the following morning.

While baseline coagulation parameters prior to PLEX were normal (APTT 34.0 s, PT 11.9 s), post-procedural laboratory tests revealed a profound depletion coagulopathy: fibrinogen levels dropped significantly to 1.35 g/L (reference: 2.00–4.00 g/L) and total protein fell by 50% (from 100 g/L to 50 g/L).

Immediate management consisted of aggressive intravenous fluid resuscitation, discontinuation of ramipril, and continuous norepinephrine infusion, which stabilized the BP to 90/70 mmHg that evening and ~110/80 mmHg the next day. To correct the hypofibrinogenemia, 2 units of group-compatible fresh frozen plasma (FFP) were transfused alongside local cold compression, improving fibrinogen levels to 1.79 g/L by the following day. Further PLEX therapy was permanently discontinued due to life-threatening hypotensive shock, multiple expanding hematomas, and severe mechanical depletion of vital plasma proteins. Hypotension was managed with fluids and norepinephrine. Due to hypofibrinogenemia and multiple hematomas, plasmapheresis was discontinued. Following treatment, symptoms improved, and IgM levels dropped significantly to 6.39 g/L. Immunosuppressive therapy with rituximab was initiated, consisting of two 1000 mg intravenous infusions two weeks apart. Crucially, rituximab was strictly scheduled and administered only after the permanent discontinuation of plasmapheresis to ensure the therapeutic monoclonal antibody was not mechanically removed by the apheresis procedure. To prevent a post-rituximab cryoglobulinemia flare, systemic glucocorticoid coverage was maintained, and close inpatient monitoring of clinical status and renal parameters was conducted. The patient tolerated the treatment well, with further stabilization of clinical and laboratory findings.

Long-term outpatient follow-up confirmed sustained clinical and laboratory stabilization; a repeat qualitative assay performed on 4 March 2025 demonstrated that circulating cryoglobulins were completely undetectable (negative), correlating with the maintenance of a low immunological disease state. Laboratory results, clinical course, and interventions are summarized in Table 1.

## 3. Discussion

The clinical presentation of HVS secondary to cryoglobulinemia in pSS represents a high-stakes medical emergency that challenges standard therapeutic paradigms [1]. While HVS is traditionally associated with monoclonal gammopathie, most notably Waldenström macroglobulinemia, its occurrence in pSS driven by mixed, polyclonal cryoglobulinemia remains an underrecognized phenomenon that demands rapid, multi-modal intervention [1,2].

Development of hyperviscosity in the context of mixed cryoglobulinemia is driven by distinct quantitative and qualitative rheological mechanisms [3]. Monoclonal IgM typically exhibits a linear concentration–viscosity relationship, but polyclonal expansion involving multiple immunoglobulin classes (IgM, IgG, and IgA) produces a profound synergistic effect [3,6]. Structurally, pentameric IgM possesses a high molecular weight (~925 kDa) and an unfavorable, large axial ratio that inherently restricts plasma layer movement [5,6]. When high concentrations of polyclonal IgM, a substantial proportion of IgM molecules possess rheumatoid factor activity, encounter circulating IgG autoantibodies, they physically cross-link to form massive, macromolecular immune complexes [1,3]. This cumulative protein load produces a disproportionate (non-linear) increase in serum viscosity as thresholds of microvascular tolerance are breached [3,6]. Further, this structural sludging is exacerbated by temperature-dependent cryoprecipitation; as ambient temperatures drop below 37 °C in acral capillary beds, these multi-isotypic immune complexes undergo physical phase separation and aggregation, compounding microvascular occlusion, driving endothelial dysfunction, and profoundly influencing the necessity for immediate cytoreductive treatment choices [1,6]. Historically, cryoglobulinemic vasculitis is characterized by the classic Meltzer’s triad: purpura, arthralgia, and weakness [1]. However, as demonstrated in this case, the clinical presentation can diverge precipitously from this classic phenotype [2]. Recent multicentre studies have confirmed that, although cutaneous purpura remains the predominant manifestation of cryoglobulinemic vasculitis in primary Sjögren’s syndrome, the presence of cryoglobulinemic vasculitis defines a severe systemic disease phenotype associated with extensive extraglandular involvement and a substantially increased risk of early B-cell lymphoma rather than isolated cutaneous disease [7]. The tissue-level deposition of immunoglobulins and complement contributes to immune-mediated skin injury. Consistent with this concept, a hospital-based study from Khartoum demonstrated that direct immunofluorescence assessment of IgA, IgG, IgM, and C3 effectively confirmed the diagnosis of autoimmune blistering diseases, highlighting the diagnostic value of combined immunoglobulin and complement evaluation in immune-mediated skin disorders [8]. Taken together, these findings support the concept that local deposition of immunoglobulins and complement reflects active immune-mediated tissue injury. In the present case, the coexistence of multiple immunoglobulin isotypes and complement deposition is consistent with an immune complex-mediated process contributing to endothelial injury, microvascular compromise, and subsequent cutaneous ischemia.

The management of cryoglobulinemic HVS necessitates a dual-phase approach: immediate physical removal of the pathogenic proteins followed by targeted immunosuppression to halt further production [2,9]. Modern consensus guidelines heavily favor B-cell depletion therapy [9,10]. Rituximab, an anti-CD20 monoclonal antibody, has emerged as the frontline agent for secondary mixed cryoglobulinemia, demonstrating superior efficacy in achieving long-term immunological remission compared to conventional glucocorticoid and cyclophosphamide regimens [2,10]. Moreover, a recent meta-analysis by Zhou et al. [10] showed that rituximab appears to be an effective treatment for cryoglobulinemic vasculitis, improving clinical outcomes and laboratory markers in most patients. Furthermore, rituximab administration can induce a transient “IgM flare”—a rapid, paradoxically sharp rise in circulating IgM levels due to cytokine release or immune complex mobilization—which can fatally exacerbate an active HVS crisis [11]. Consequently, biological therapies cannot be used as standalone monotherapy during the acute, rheological phase [2,10,11]. In the case of our patient, rituximab was administered as two 1000 mg intravenous infusions 14 days apart. Although some experts prefer the four-weekly 375 mg/m^2^ regimen due to a potentially lower flare risk in cryoglobulinemia, the 2 × 1000 mg protocol was chosen based on specific clinical considerations outlined: first, it represents the regimen used in the only randomized controlled trial (RCT) in cryoglobulinemic vasculitis [12]; second, the KDIGO 2022 guidelines list both schedules as equivalent options [13]; and finally, the patient’s limited compliance made an incomplete four-weekly induction a greater clinical risk than the theoretical flare risk. Choosing this condensed fixed-dose approach ensured full and rapid treatment completion while successfully establishing long-term B-cell depletion and clinical stabilization [1,2,9]. Considering the role of Plasmapheresis and Long-Term Immunological Monitoring, in the acute phase of HVS, Therapeutic Plasma Exchange (TPE) remains the gold standard intervention [14]. Because approximately 80% of IgM is sequestered intravascularly, TPE is highly effective, capable of reducing IgM levels and serum viscosity by up to 50–80% after a few sessions [3,10,14]. According to the updated American Society for Apheresis (ASFA) 2023 guidelines, TPE is designated as a Category I or II recommendation for acute hyperviscosity and severe cryoglobulinemic manifestations, serving as an unmatched modality to rapidly restore microvascular perfusion, reverse ischemic retinopathy, and mitigate neurological deterioration [15]. Beyond acute stabilization, the long-term prognosis of pSS-associated cryoglobulinemic HVS depends heavily on structured, longitudinal surveillance of surrogate immunologic markers [1,2]. Clinicians must shift focus from reactive crisis management to proactive monitoring, where tracking serum cryocrit percentages, rheumatoid factor activity, and complement consumption (specifically C4 and C3 depletion) serves as a critical early-warning system [2,7]. As demonstrated by our patient’s clinical course, achieving sustained clinical and immunological remission—marked by the complete clearance of circulating cryoglobulins post-rituximab therapy—underscores that strategic B-cell depletion can successfully reset the humoral immune response. Next, as it was presented in other paper that long-term plasmapheresis, used in combination with thalidomide and dexamethasone, may be an effective and well-tolerated treatment for refractory cases of type I cryoglobulinemia, leading to healing of skin lesions and control of organ complications [16]. Ultimately, our case highlights that integrating routine, active screening for subclinical cryoglobulinemia in asymptomatic pSS cohorts with extreme polyclonal hypergammaglobulinemia is vital to prevent precipitous, life-threatening microvascular failure before an irreversible rheological crisis establishes [2,7]. Our previous analysis of 21 patients with secondary HLH also identified hypofibrinogenemia as a common laboratory feature of severe systemic hyperinflammation, emphasizing the importance of careful coagulation monitoring when interpreting coagulation abnormalities in critically ill patients with immune-mediated diseases [17].

A limitation of this case report Is the lack of objective, quantitative data for serum viscosity (in centipoise) and cryocrit percentage, which could not be obtained due to institutional unavailability of these tests. Nevertheless, the diagnosis of hyperviscosity syndrome was firmly established based on compelling clinical features, characteristic ophthalmologic findings, and severe hypergammaglobulinemia. Management of cryoglobulinemia-associated hyperviscosity syndrome requires prompt intervention to reduce circulating cryoglobulin levels and restore tissue perfusion. Plasmapheresis is widely recognized as the elective therapy for acute hyperviscosity, enabling rapid removal of pathogenic proteins and immune complexes [3,4,15]. The procedure is indicated in severe or refractory cases and may be used alone or as an adjunct to immunosuppressive or disease-targeted therapies [1,2,10,15]. Recent advances, such as double-filtration plasmapheresis, allow for selective removal of cryoprecipitating proteins while preserving essential plasma components [5,10,15]. Despite the lack of randomized controlled trials, observational data and expert consensus support the efficacy of plasmapheresis in rapidly ameliorating symptoms and preventing irreversible organ damage in cryoglobulinemia-associated hyperviscosity syndrome [2,15].

## 4. Conclusions

In conclusion, this case underscores that severe, life-threatening HVS can be actively driven by an extreme polyclonal increase in immunoglobulin, such as that observed in primary Sjögren’s syndrome, rather than being exclusively limited to traditional monoclonal gammopathies. Clinicians must maintain a high index of suspicion for this atypical rheological presentation when managing patients with profound hypergammaglobulinemia who exhibit acute microvascular or neurological decline and prompt clinical decision-making is essential.

## Figures and Tables

**Table 1:** Timeline linking laboratory results, clinical course, and interventions.

Time Point/Date	Clinical Manifestations & Symptoms	Medical Interventions & Procedures	Key Laboratory Findings
**Pre-admission** *(History)*	12-year history of Sjögren’s syndrome treated with systemic glucocorticosteroids and chloroquine.	Urgent referral to the Department of Rheumatology and Immunology.	Interstitial kidney disease, polyclonal hypergammaglobulinemia, present cryoglobulins
**25 March 2024** *(Baseline/Admission)*	New-onset headache, lower extremity pain, Raynaud’s phenomenon, reduced exercise tolerance, recurrent epistaxis, facial and upper extremity petechial rash, distal extremity cooling/cyanosis, livedo reticularis.	Hospital admission. Head CT (mastoiditis), chest/abdominal/pelvic CT (no lymphadenopathy/organomegaly), ophthalmologic exam (retinopathy), bone marrow biopsy.	**Total protein:** 100 g/L **IgM:** 61.3 g/L *(by timeline)*/41 g/L *(by quantitative test)* **IgG:** 40 g/L **IgA:** 23 g/L **Coagulation:** APTT 34.0 s, PT 11.9 s (normal) **Cryocrit/Viscosity:** Not performed (unavailable) **Other:** Hb 10.1 g/dL, Creatinine 131.8 µmol/L, eGFR 38 mL/min/1.73 m^2^, cryoglobulins (+), free kappa 222 mg/L.
**28–29 March 2024** *(Therapy Initiation)*	Clinically stable; no immediate adverse events reported during the first two sessions.	**TPE Session 1:** 2000 mL plasma volume exchange. **TPE Session 2:** 2500 mL plasma volume exchange. *(Replacement: 5% human albumin; Anticoagulation: UFH; Pre-medication: methylprednisolone, electrolytes)*.	Routine parameters monitored; no specific inter-procedural immunoglobulin levels reported.
**29 March 2024** *(Daytime to Night)*	**Daytime:** Completion of the third TPE session (2500 mL). **+20 min post-TPE:** Sudden hemodynamic collapse, critical hypotension (BP 50/30 mmHg, HR 90 bpm, SaO_2_ 92%), no loss of consciousness **Night (21:38):** Onset of bleeding complications, localized left trochanteric hematoma.	**Emergency Interventions:** Aggressive intravenous fluid resuscitation Continuous norepinephrine infusion. Discontinuation of ramipril. BP stabilized to ~90/70 mmHg by evening.	Baseline coagulation was normal prior to PLEX. Post-procedural blood samples collected overnight revealed a profound depletion/dilutional coagulopathy.
**30 March 2024** *(Clinical Crisis & Protocol Modification)*	**Morning:** Multiple massive soft-tissue hematomas expanding on both arms and the left hip.	**Protocol Modification & Rescue:** **Permanent discontinuation of PLEX** (due to shock, expanding hematomas, protein depletion). Transfusion of **2 units of group-compatible fresh frozen plasma (FFP)**. Local cold compression Vasopressor adjustments (BP stabilized to ~110/80 mmHg).	**Fibrinogen:** dropped significantly to **1.35 g/L** (severe hypofibrinogenemia; reference: 2.00–4.00 g/L). **Total Protein:** fell by ~50% (from 100 g/L down to **50 g/L**).
**31 March 2024 & beyond** *(Resolution & Outcome)*	Rapid clinical resolution of hyperviscosity-related symptoms (retinopathy, neurological symptoms, and skin ischemia).	Vasopressors successfully tapered off.	**Fibrinogen:** improved to **1.79 g/L** (post-FFP transfusion). **IgM:** significantly reduced to **6.39 g/L** (~85–90% reduction from baseline).
**Post-stabilization** *(Maintenance)*	Well tolerated, leading to sustained clinical and laboratory stabilization.	**Targeted B-cell Immunosuppression:** Induction with **Rituximab**, administered as two 1000 mg intravenous infusions two weeks apart.	Sustained reduction in IgM levels and stabilization of renal functions during ambulatory follow-ups. Cryoglobulins negative.

## Data Availability

No new datasets were generated or analyzed during this study. Data supporting the findings of this case report are not publicly available due to patient privacy considerations.
